# Clinical Application of Cone Beam Computed Tomography of the Rabbit Head: Part 2—Dental Disease

**DOI:** 10.3389/fvets.2017.00005

**Published:** 2017-01-30

**Authors:** G. G. Riggs, Derek D. Cissell, Boaz Arzi, David C. Hatcher, Philip H. Kass, Amy Zhen, Frank J. M. Verstraete

**Affiliations:** ^1^Dentistry and Oral Surgery Service, William Pritchard Veterinary Medical Teaching Hospital, School of Veterinary Medicine, University of California, Davis, CA, USA; ^2^Department of Surgical and Radiological Sciences, School of Veterinary Medicine, University of California, Davis, CA, USA; ^3^Diagnostic Digital Imaging Center, Sacramento, CA, USA; ^4^Department of Population Health and Reproduction, School of Veterinary Medicine, University of California, Davis, CA, USA

**Keywords:** cone beam computed tomography, rabbit, *Oryctolagus cuniculus*, dental disease, diagnostic imaging

## Abstract

Domestic rabbits are increasing in popularity as household pets; therefore, veterinarians need to be familiar with the most common diseases afflicting rabbits including dental disease. Current diagnostic approaches include gross oral examination, endoscopic oral examination, skull radiography, and computed tomography (CT). Cone beam computed tomography (CBCT), a new oral and maxillofacial imaging modality that has the capability to produce high-resolution images, has not yet been described for use in evaluating dental disease in rabbits. A total of 15 client-owned rabbits had CBCT, oral examination, dental charting, and dental treatment performed under general anesthesia. Images were evaluated using transverse and custom multiplanar (MPR), 3D, and panoramic reconstructed images. The CBCT findings were grouped into abnormalities that could be detected on conscious oral examination vs. abnormalities that could not be detected by conscious oral examination. Potential associations between the two categories were examined by pairwise Fisher’s exact test with statistical significance determined by *P* < 0.05. The most common findings identified on CBCT images were periodontal ligament space widening (14/15), premolar and molar malocclusion (13/15), apical elongation (13/15), coronal elongation (12/15), inflammatory tooth resorption (12/15), periapical lucency (11/15), moth-eaten pattern of osteolysis of the alveolar bone (9/15), ventral mandibular border contour changes (9/15), and missing teeth (8/15). Of the CBCT abnormalities likely to be observed on oral examination, coronal elongation (detectable on oral examination) was significantly associated with apical elongation (*P* = 0.029). There were no other significant associations between CBCT findings that are also clinically detectable and CBCT findings that are not be detectable on oral examination. This suggests that pathology often exists that is not apparent upon oral examination. This study establishes the common CBCT findings associated with dental disease in rabbits and demonstrates the feasibility of this technology to diagnose and plan treatment in dental disorders in this species.

## Introduction

Domestic rabbits are increasing in popularity as household pets. Therefore, veterinarians need to be familiar with the most common diseases afflicting rabbits including dental disease. The continuous growth of rabbit teeth make them susceptible to a plethora of oromaxillofacial and dental abnormalities such as malocclusion, coronal and apical elongation, sharp dental points, lingual and buccal mucosal ulcers, facial abscesses, endodontal disease, diastema formation, feed impaction, and periodontal disease (1–6). Physiologic wear due to mastication and occlusal contact compensates for the continuous growth of teeth; however, malocclusion and dental disease are common among domestic rabbits (7). The causes for dental disease and malocclusion may include trauma, congenital abnormalities, improper diet, and preexisting malocclusion. Clinical signs observed are non-specific and may include lethargy, anorexia, weight loss, excessive salivation, facial swelling, and inability to close the mouth, decreased grooming, and decreased fecal output due to gastrointestinal stasis (4, 8, 9). Depending on the teeth affected, ocular and nasal discharge as well as exophthalmos can occur (4, 8, 9).

Due to the small oral cavity of rabbits and their tendency to become stressed with handling, it is challenging to examine their teeth on conscious oral examination. Importantly, the findings on oral examination while the rabbit is awake may not be representative of the severity of the dental disease. Besides gross oral examination, diagnostic approaches include endoscopic oral examination, skull radiography, and computed tomography (CT) (1, 4, 8–10). Skull radiography has disadvantages such as superimposition of dental structures and inability to isolate dental lesions (9, 11). Traditional CT is currently the imaging modality of choice for precise diagnosis of dental diseases; however, it has a few disadvantages such as relatively thick slices and high costs. In contrast, cone beam computed tomography (CBCT) is an oral and maxillofacial imaging modality that has the capability to produce high-resolution images (12–14). CBCT is approximately one-half the cost of conventional CT. The improved spatial resolution, lower cost, and comparable or faster scan times of CBCT compared to conventional CT make CBCT an attractive diagnostic tool for evaluating small animals with dental disease.

In part one of this two-part study, we described the normal anatomic features of the dentition and surrounding maxillofacial structures in healthy rabbits by means of CBCT and conventional CT. The visibility of relevant dental and anatomic features (pulp cavity, germinal center, tooth outline, periodontal ligament) were scored and compared between conventional CT and CBCT. In general, it was found that CBCT was superior to conventional CT when imaging the dentition. An important finding was that the periodontal ligament was significantly (*P* < 0.01) more visible on CBCT than on conventional CT (12).

The objective of part two of this study was to provide insight into the possibilities and limitations of CBCT and image manipulation for diagnosis of dental pathology in rabbits. We hypothesize that CBCT will detect dental pathology that cannot be observed or predicted by physical and oral examinations alone.

## Materials and Methods

### Criteria for Selection of Cases

This is a prospective study using client-owned rabbits that were treated by the Veterinary Medical Teaching Hospital (VMTH), Dentistry and Oral Surgery Service and Companion Exotic Animal Medicine & Surgery Service for dental disease. This study was conducted with approval of the Institutional Animal Care and Use Committee (protocol 18050) and the Clinical Trials Review Board, University of California, Davis. All owners signed an informed consent form. Inclusion criteria for this study included rabbits affected by dental disease, such as abscessation, malocclusion, and tooth elongation, based on presenting complaint and oral examination findings. Complete blood count and serum biochemistry were used to screen for systemic illness. Rabbits with evidence of concurrent diseases (e.g., renal failure) that posed an increased anesthetic risk were not included in the study. Rabbits with maxillary or mandibular swellings that were later diagnosed or suspected to be neoplastic in nature were excluded from this study. Rabbits with clinical signs potentially referable to dental, but without oral examination or CBCT evidence of dental disease were excluded from analysis.

### Procedures

All rabbits underwent general anesthesia and were scanned in a NewTom 5G CBCT with various field of view (FOV) settings: 10 rabbits were scanned with FOV = 15 cm × 12 cm; three rabbits with 12 cm × 8 cm; and two rabbits with 18 cm × 16 cm. Fourteen of the scans collected 0.2-mm thick slices, whereas 0.25-mm slices were collected for rabbit #9 due to the larger FOV required to image the entire skull of this rabbit. Manipulation and evaluation of the images was performed using Anatomage InVivo 5 software (San Jose, CA, USA). Treatment and additional diagnostic tests were dependent on diagnosis and included occlusal adjustment, dental extractions, bacterial culture and sensitivity, and marsupialization of abscesses.

### Imaging Evaluation and Statistics

All CBCT scans were evaluated for the presence or absence of incisor malocclusion, premolar and molar malocclusion, presence of sharp dental points, coronal elongation, apical elongation, ventral mandibular osseous contour changes, periodontal ligament space widening, periapical lucency, moth-eaten pattern of osteolysis of the alveolar bone (maxillary and/or mandibular), inflammatory tooth root resorption, tooth fragmentation secondary to tooth root resorption, gas opacities within the soft tissue or bone, fractured teeth, missing teeth, and supernumerary teeth. When malocclusion of the incisor or premolar/molar teeth was present, it was recorded if the malocclusion was symmetric or asymmetric between the right and left sides of the skull. Apical or coronal elongation was determined from the panoramic images based on the methods described by Böhmer and Crossley (15). External inflammatory tooth resorption was defined as the loss of dental tissues adjacent to areas of loss of alveolar bone secondary to inflammatory conditions (i.e., endodontal or periodontal disease), and internal inflammatory tooth resorption was defined as an oval-shaped enlargement within the tooth due to loss of dental substance (16). A periapical lucency rather than periapical abscess was used. A periapical lucency was defined as a well-defined lucency around the germinal center and associated reserve crown of the teeth.

The severity of the dental disease was determined to be mild, moderate, or severe based on all findings evaluated. When most of the teeth were present, tooth integrity was largely maintained, the maxillary and mandibular bones were largely unaffected, individually diseased teeth only affected the immediately adjacent teeth rather than the entire quadrant, and the teeth maintained a relatively normal relationship to one another, this was categorized as mild dental disease. When more teeth were missing, less than half of the teeth started to lose integrity, one of the quadrants was affected by moderate moth-eaten osteolysis of the alveolar bone, individually diseased teeth started to affect the entire quadrant, the space between teeth began to widen, and the steps in the occlusion started to become more pronounced, this was categorized as moderate dental disease. In the severe dental disease category, up to four quadrants of teeth were severely affected by moth-eaten osteolysis. Moth-eaten osteolysis of the alveolar bone may have been so pronounced that pathologic fracture of the mandible was possible. The inflammatory tooth resorption affected more than half of the teeth and the teeth were starting to fragment, making them difficult to identify or distinguish from one another.

Data were grouped into abnormalities that could be detected on oral examination vs. abnormalities that could not be detected on oral examination. Associations between these categorical variables were examined by pairwise Fisher’s exact test (Stata IC/13.1, StataCorp LP, College Station, TX, USA) with statistical significance determined by *P* < 0.05.

## Results

### Patient Population

Fifteen rabbits met the criteria for inclusion. Patients included seven Holland lop or lop mixes, three Dutch or Dutch mixes, two mini Rex, and one each of California mix, lionhead mix, and a Netherland dwarf. The age of the rabbits ranged from 1.8 to 12.2 years (mean = 5.9, median = 5) with two of the rabbits being adults of unknown age. The sex distribution was eight males and seven females. The reproductive status was two intact males, one intact female, six neutered males and six spayed females. The body weight of the rabbits ranged from 1.1 to 3.5 kg (mean = 2.2, median = 2.2) (Supplementary Table 1).

### Historical and Physical Examination Findings

Most rabbits were presented for known or suspected dental disease with dental-related clinical signs of durations ranging from 0.2 to 64.4 months (mean = 13.3, median = 3.8). The known duration of clinical signs for the rabbits with mild dental disease was 0.2 to 11.4 months (mean = 4.5, median = 3.3), moderate dental disease was 0.3 to 64.4 months (mean = 14.4, median = 2.0), and severe dental disease was 3.0 to 62.1 months (mean = 18.3, median = 4.1). The most common clinical signs at presentation were hyporexia (8/15), ocular discharge (7/15), nasal discharge (6/15), and sneezing (3/15). Few rabbits had decreased fecal output (1/15) and lethargy (2/15). No rabbits were presented with ptyalism, but two rabbits had a history of ptyalism. Six of 15 rabbits have had a previous history of hyporexia. Recent dental extractions were reported in 1 of 15 rabbits. Occlusal adjustments within the previous 7 months were reported in 4 of 15 rabbits. Nine of the 15 rabbits had occlusal adjustments in their lifetime. The majority of the rabbits were already being administered medications for treatment of oral pain and infection such as meloxicam (10/15), tramadol (5/15), and various antibiotics (8/15) (Supplementary Table 1).

On physical examination, 10 of 15 rabbits had swellings, masses, or osseous protuberances palpable on the ventral mandible, whereas only 2 of 15 rabbits had similar findings on the maxilla. Three of 15 rabbits had exophthalmos. Common additional findings in rabbits with dental disease were pododermatitis (11/15) and matted fur around the head, neck, and forefeet (9/15). One rabbit was emaciated with a body condition score (BCS) of 1/9, one rabbit was under conditioned with a BCS 3/9, seven rabbits had ideal body conditions of 4–5, and six rabbits were over conditioned with BCS 6/9 and above.

### Diagnostic Imaging Findings

Cone beam computed tomography examination of the rabbits in this study revealed a variety of findings in all cases that may not have been as easily identified on skull radiography or conventional CT. The most common findings were periodontal ligament space widening (14/15), premolar and molar malocclusion (13/15), apical elongation (13/15), coronal elongation (12/15), inflammatory tooth resorption (12/15), sharp dental points (12/15), periapical lucency (11/15), a pattern of moth-eaten osteolysis of the alveolar bone (9/15), ventral mandibular border contour changes (9/15), and missing teeth (8/15). Four rabbits were determined to have mild dental disease, five had moderate dental disease, and six had severe dental disease based on CBCT evaluation. The CBCT findings organized by severity of dental disease are available in Table 1.

**Table 1:** **Cone beam computed tomography findings in mild, moderate, and severe dental disease**.

Category evaluated	Mild dental disease	Moderate dental disease	Severe dental disease	Total rabbits affected
Incisor malocclusion	1/4	1/5	2/6	4/15
Premolar and molar malocclusion	3/4	4/5	6/6	13/15
Coronal elongation	2/4	4/5	6/6	12/15
Apical elongation	2/4	5/5	6/6	13/15
Ventral mandibular border osseous contour changes	1/4	2/5	6/6	9/15
Periodontal ligament space widening	3/4	5/5	6/6	14/15
Periapical lucency	1/4	5/5	5/6	11/15
Moth-eaten osteolysis of the alveolar bone	0/4	4/5	5/6	9/15
Inflammatory tooth resorption	1/4	5/5	6/6	12/15
Tooth fragmentation	0/4	1/5	5/6	6/15
Fractured teeth	1/4	1/5	1/6	3/15
Missing teeth	1/4	2/5	5/6	8/15
Sharp dental points	2/4	4/5	6/6	12/15

**Figure 1:**
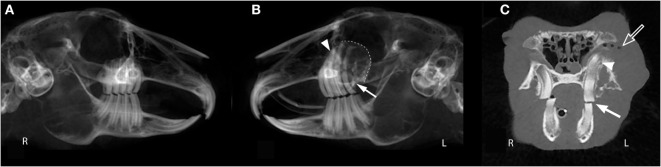
**Oral examination vs. cone beam computed tomography findings**. This panoramic image of rabbit #10 **(A,B)** demonstrates that occlusion can appear normal *via* oral examination despite the presence of dental pathology including severe apical elongation with inflammatory root resorption (white arrow head), moth-eaten osteolysis of the left maxillary alveolar bone (dotted line), and a missing left maxillary third molar tooth (white arrow). The transverse image of rabbit #10 **(C)** shows a normal 10° occlusal angle on the right and a flat occlusal angle on the left (white arrow). Other findings in this image include apical elongation (white arrow head), gas opacities in the retrobulbar space (open arrow), and a pattern of moth-eaten osteolysis of the alveolar bone at the buccal aspect of the left maxillary first molar tooth.

Thirteen of 15 rabbits had premolar and molar malocclusion, and of these rabbits, 7 had symmetric and 6 had asymmetric malocclusion and dental pathology. Four rabbits had incisor malocclusion; three of these four rabbits also had premolar and molar malocclusion. Only one rabbit had incisor malocclusion without premolar and molar malocclusion. All four rabbits with incisor malocclusion had symmetric malocclusion. One rabbit had normal occlusion (Figures 1A,B), but had other CBCT findings including periodontal ligament space widening, periapical lucency, moth-eaten osteolysis of the alveolar bone, apical elongation, inflammatory root resorption, and a missing tooth. Incisor occlusion was not associated with premolar/molar malocclusion (*P* = 0.47).

**Figure 2:**
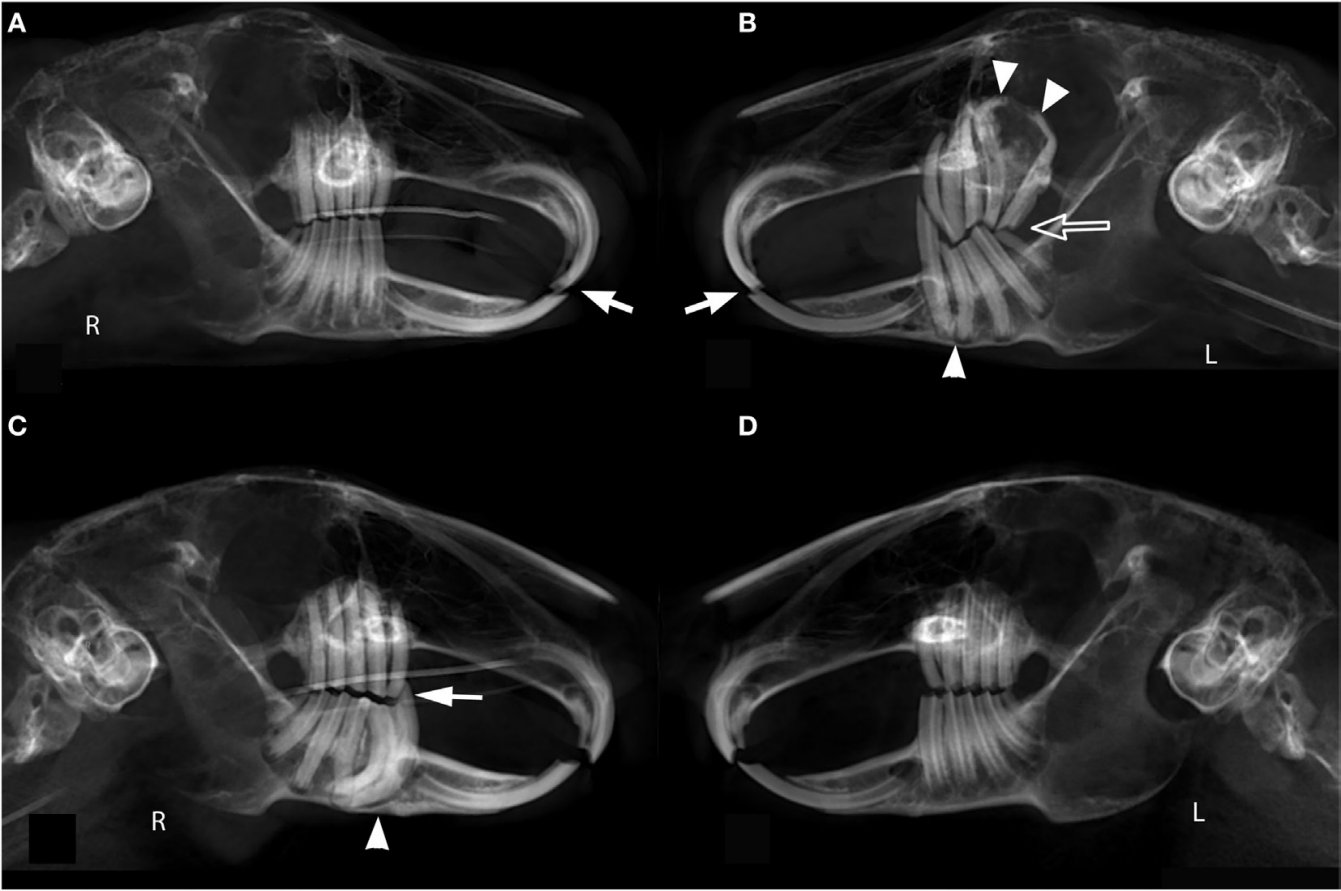
**Symmetry of occlusion**. The panoramic image of rabbit #9 **(A,B)** shows symmetric incisor malocclusion (white arrows) and asymmetric premolar and molar teeth malocclusion with sharp dental points and coronal tooth elongation (open arrow). The left maxilla and mandible show apical tooth elongation, severe malangulation of the reserve crowns of the maxillary first and second molar teeth (white arrow heads), and regional moth-eaten osteolysis of the alveolar bone of the maxilla. The panoramic image of rabbit #2 **(C,D)** depicts an asymmetric premolar and molar malocclusion secondary to a supernumerary right mandibular third premolar tooth with associated apical elongation and periodontal ligament space widening (white arrow head). Additionally, there is coronal elongation and a sharp dental point of the rostral most tooth (white arrow).

Eleven of the 13 rabbits with premolar and molar malocclusion were due to coronal elongation. One other rabbit had coronal elongation of only the incisor teeth causing incisor malocclusion. One rabbit had a supernumerary right mandibular third premolar tooth with coronal elongation (Figures 2C,D). Coronal elongation was not associated with incisor malocclusion (*P* = 0.516) or premolar and molar malocclusion (*P* = 0.371).

Of the CBCT abnormalities likely to be observed on oral examination, coronal elongation was significantly associated with apical elongation (*P* = 0.029), all other comparisons of oral examination findings vs. abnormalities not detectable on oral or physical examination were not significant (Table 2).

**Table 2:** ***P*-values of categories detectable on the oral examination and physical examination (row headers) and categories detectable on cone beam computed tomography (column headers)**.

Category	Apical elongation	Periodontal ligament space widening	Periapical lucency	Moth-eaten osteolysis of the mandibular alveolar bone	Moth-eaten osteolysis of the maxillary alveolar bone	Moth-eaten osteolysis of the maxillary or mandibular alveolar bone	Inflammatory tooth resorption	Tooth fragments
Incisor malocclusion	1	1	0.516	1	0.282	0.604	0.516	0.604
Premolar and molar malocclusion	1	1	1	1	1	1	1	0.486
Coronal elongation	0.029^a^	0.200	0.154	0.569	1	0.525	0.081	0.229
Ventral mandibular border contour changes	0.143	0.400	1	0.315	0.608	0.622	0.525	0.287
Missing teeth	1	1	1	0.619	0.315	1	0.569	0.608
Exophthalmos	0.371	1	1	1	0.569	1	0.516	1
Sharp dental points	0.371	1	1	0.569	1	0.525	0.516	0.229

*^a^Statistical significance on Fisher’s exact pairwise comparison*.

**Figure 3:**
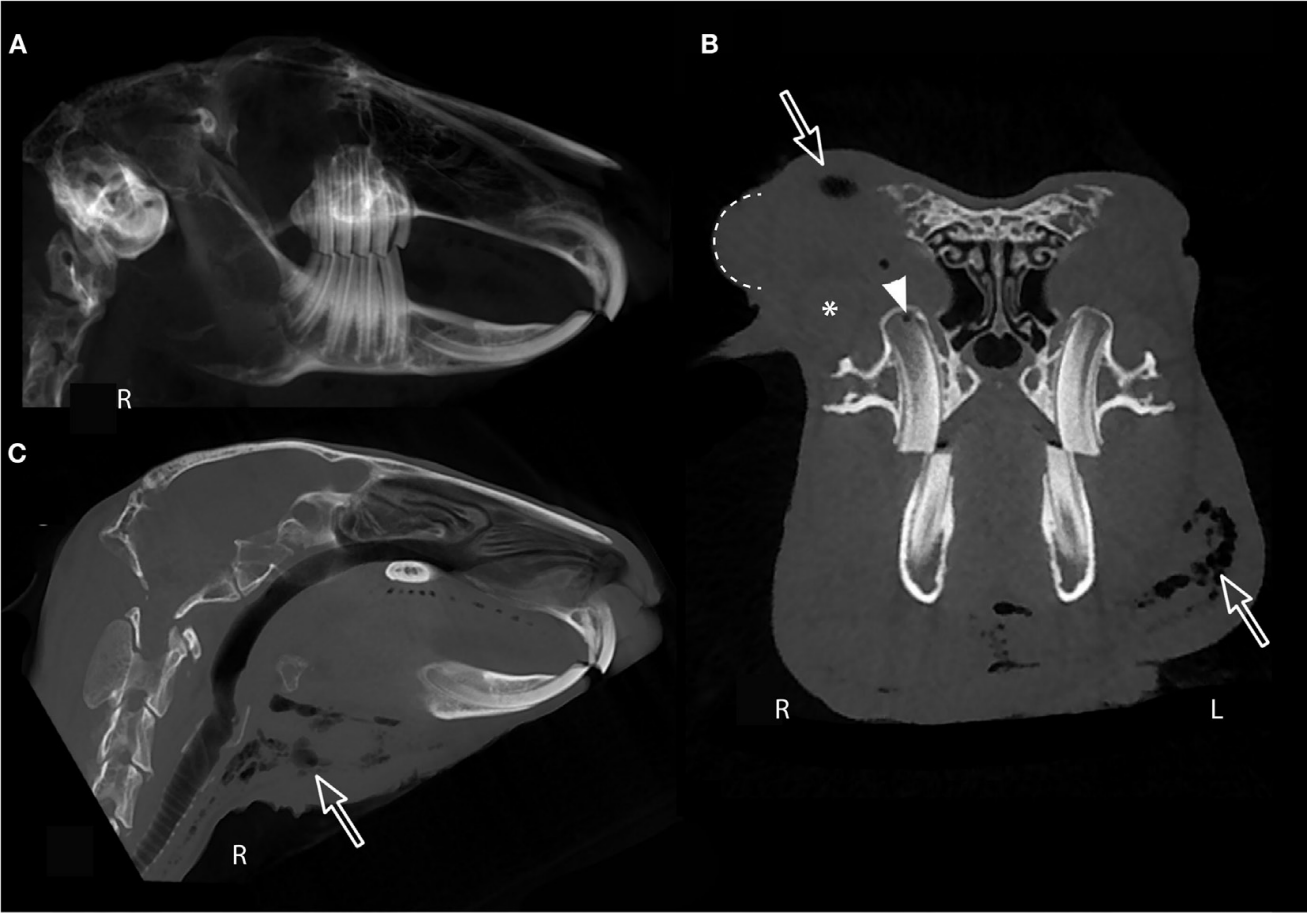
**Cone beam computed tomography soft tissue evaluation**. **(A)** Right panoramic image of rabbit #5 showing normal incisor, premolar, and molar occlusion. Transverse image immediately rostral to the globe **(B)** and median sagittal multiplanar image **(C)** of the same rabbit showing soft tissue submandibular swelling, right-sided periocular swelling and chemosis of the nictitating membrane (dotted line). The zygomatic salivary gland is indicated by an asterisk (*). Gas opacities are present in the submandibular region and retrobulbar space (open arrows) and within the right maxillary first molar germinal center (white arrow head).

Other findings included gas opacities within the soft tissue or bone in 3 of 15 rabbits (Figure 3). Twelve rabbits had gas opacities near the medial canthus of the eyes, corresponding to the entrance of the nasolacrimal duct and gas trapped under the eyelids. Three rabbits had mineralization of the ventral nasal concha.

Thirteen rabbits had ventral mandibular border changes that could potentially be palpated on physical examination based on CBCT imaging. Nine of these 13 rabbits with ventral mandibular border changes had apical elongation, but this association was not significant (*P* = 0.143). Coronal elongation was significantly associated with ventral mandibular border changes (*P* = 0.044).

**Figure 4:**
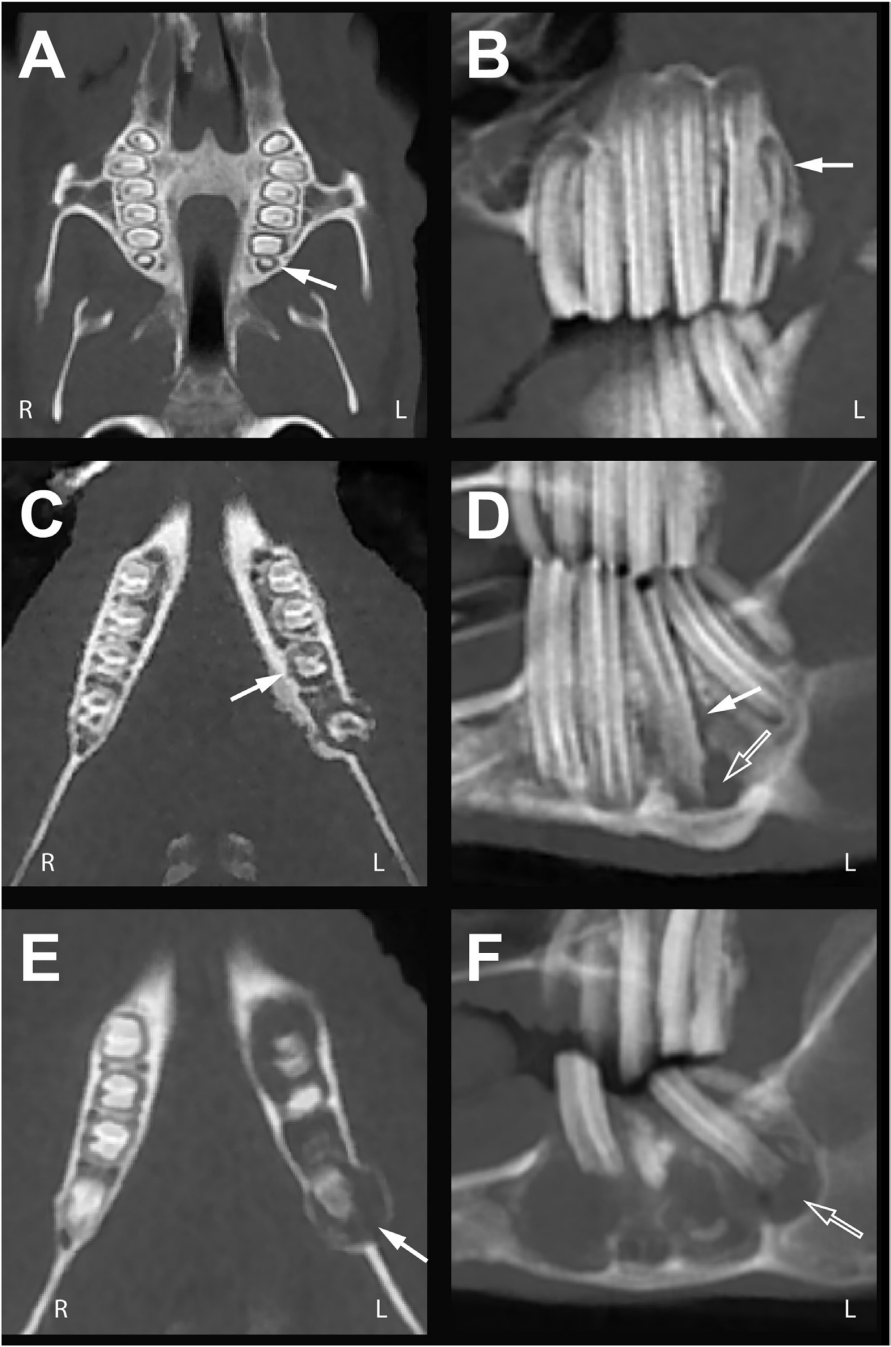
**Mild, moderate, and severe periodontal ligament space widening**. Dorsal multiplanar (MPR) (left column) and sagittal MPR (right column) cone beam computed tomography images of rabbits with multiple teeth exhibiting varying degrees of periodontal ligament space widening. **(A,B)** In rabbit #8, periodontitis of the left maxillary third molar tooth is visible as subtle periodontal ligament space widening (white arrows). **(C,D)** In rabbit #1, periodontitis has progressed at the left mandibular first molar tooth from widening of the periodontal ligament space (white arrows) to formation of periapical lucency (open arrow). **(E,F)** In rabbit #7, periodontal ligament space widening of multiple teeth (white arrow) is contiguous with the periapical lucency and pattern of moth-eaten osteolysis (open arrow). Expansion and osteolysis of the alveolar bone is evident in this rabbit.

All but one rabbit had evidence of periodontal ligament space widening. Three of these 14 rabbits only had periodontal ligament space widening, whereas the other 11 also had periapical lucencies. Of the 11 rabbits with periodontal ligament space widening and periapical lucencies, nine of them also had a pattern of moth-eaten osteolysis of the alveolar bone (Figure 4).

**Figure 5:**
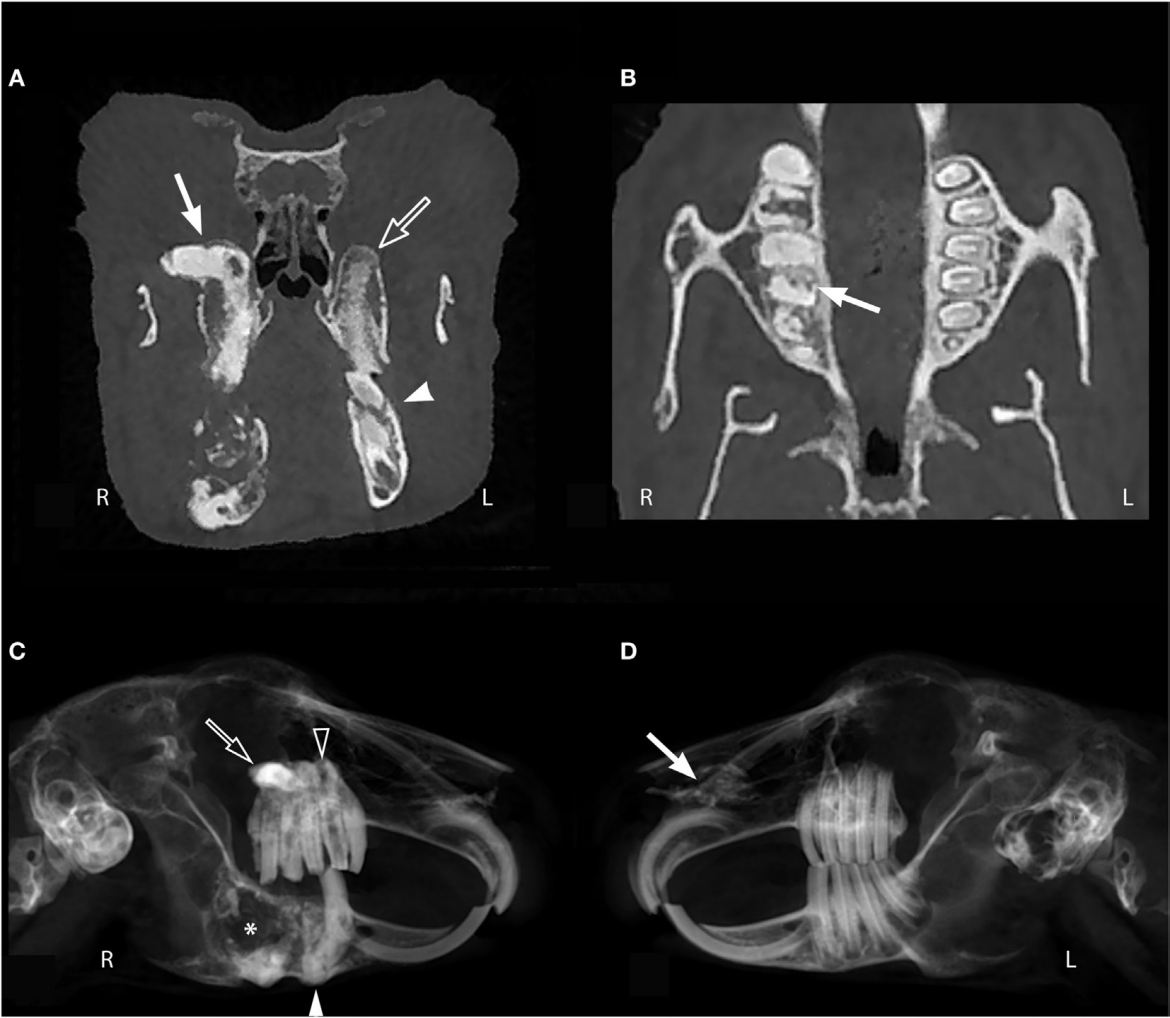
**Inflammatory tooth resorption**. **(A)** Irregular margins and lucency indicative of resorption of the right maxillary first molar tooth (white arrow). A normal tooth that is not fully within the plane of the image (open arrow) can appear lucent and irregular as though it has resorption due to volume average artifact. Similarly, if the teeth are angled, the transverse image may show the clinical crown of one tooth, but the reserve crown of an adjacent tooth (white arrow head), creating the artifactual appearance of tooth resorption or fragmentation. **(B)** Tooth resorption may be more evident on dorsal multiplanar images (white arrow). **(C,D)** In these panoramic images of rabbit #11, tooth resorption is severely affecting the right premolar and molar teeth, but not the left. Multiple mandibular teeth are absent from the right mandible and there is resorption, coronal elongation, and apical elongation of the third premolar tooth (white arrowhead) with severe osteolysis of the mandible (*). Additionally, there is elongation of the right maxillary premolar and molar teeth with resorption of the third premolar tooth (open arrow head). Increased opacity is visible superimposed on the apices of the right maxillary first and second molars (open arrow) due to elongation and malangulation of the reserve crowns. Mineralization of the left nasal cavity (white arrow) was an incidental finding.

All nine rabbits with a pattern of moth-eaten osteolysis of the alveolar bone also had inflammatory tooth resorption (Figure 5). An additional three rabbits had inflammatory tooth resorption without moth-eaten osteolysis of the alveolar bone. Two of these rabbits had periodontal ligament space widening and periapical lucencies, whereas the remaining one rabbit only had periodontal ligament space widening. Periapical lucency was associated with moth-eaten osteolysis of the alveolar bone (*P* = 0.011) and with inflammatory root resorption (*P* = 0.009). Moth-eaten osteolysis of the alveolar bone (*P* = 0.044) and apical elongation (*P* = 0.029) were also associated with inflammatory root resorption. Of the 12 total rabbits with inflammatory tooth root resorption, six of them had such severe resorption that the teeth fragmented. Three of the 15 rabbits had true tooth fractures. Eight of the 15 rabbits already had missing teeth.

## Discussion

The present study is the first to demonstrate the feasibility and yield of CBCT in evaluating maxillofacial features and dentition in rabbits with dental disease. The results of this study support our hypothesis that CBCT will detect dental pathology that cannot be observed or predicted by physical and oral examinations alone. Additionally, abnormalities detected on physical and oral examinations are not necessarily associated with the presence of dental abnormalities, such as periodontal ligament space widening, periapical lucencies, moth-eaten osteolysis of the alveolar bone, or inflammatory tooth resorption that will influence the choice of treatment. Therefore, advanced imaging is required when dental disease is suspected to fully characterize the extent and nature of the dental pathology and inform appropriate therapy.

The main take-home points include the following:

- The known duration of the dental disease did not necessarily match the severity of the dental disease.
- Malocclusion may be symmetric or asymmetric and the associated clinical signs may not match the severity of the disease.
- CBCT scan is optimized to image the osseous maxillofacial features rather than the soft tissues, but evaluation of the soft tissues is still possible on transverse and custom MPR images. The 3D reconstruction and panoramic images give the clinician a “global feel” of the dentition and maxillofacial features, but soft tissue findings can be overlooked with these views.
- The periodontal ligament is well visualized on transverse and custom MPR images. Widening of the periodontal ligament can progress to periapical lucency and then to a pattern of moth-eaten osteolysis of the alveolar bone.
- Tooth resorption can be difficult to evaluate solely from transverse images and is easier to evaluate on panoramic, sagittal MPR, and dorsal MPR images.

There was a relatively even distribution of rabbits with mild (four rabbits), moderate (five rabbits), and severe dental disease (six rabbits). The known duration of the dental disease did not necessarily match the severity of the dental disease. For example, rabbits #12 and #14 had severe dental disease, but the duration of known clinical signs was 3.8 and 4.3 months, respectively. Thus, it is likely that rabbits with severe or moderate dental disease, and short duration of clinical signs, may have had dental disease for longer than the duration clinical signs were noticed by the client.

Incisor malocclusion is more visually evident to the client, but it is important for the veterinarian to also evaluate the premolar and molar teeth when performing an oral evaluation. The current literature states that incisor malocclusion is common in pet rabbits and that incisor malocclusion without premolar and molar abnormalities may be relatively rare, especially in older rabbits (4, 17). In the present study, more rabbits (13/15) had premolar and molar malocclusion than had incisor malocclusion (4/15). Only one rabbit had incisor malocclusion in the absence of premolar and molar malocclusion. Previous literature supports that if incisor malocclusion occurs as an isolated entity at an early age, it is probably due to maxillary brachygnathia, which has a genetic origin (4, 18–20). The one rabbit with isolated incisor malocclusion was a 3.9-year-old, female spayed, Holland lop rabbit. She was one of the four youngest rabbits in this study, which may indicate that her incisor malocclusion was genetic in origin, but evaluation of her CBCT scan does not show maxillary brachygnathia. Malocclusion and fracture of the right maxillary first incisor tooth may have been secondary to trauma or iatrogenic trauma associated with incisor trimming, as she was not reported to have incisor malocclusion in the years previous. There were 10 rabbits with premolar and molar malocclusion that did not have incisor malocclusion. Our data suggest that incisor malocclusion and premolar and molar malocclusion are not associated (*P* = 0.47). Once explanation of this finding is that these rabbits were having incisor trimmings without proper premolar and molar adjustments, thereby skewing our data. In contrast, the current literature states that incisor malocclusion may be secondary to, or occur concomitantly with, premolar and molar malocclusion. Additionally, incisor malocclusion that prevents normal mastication may lead to premolar and molar malocclusion (17). It is also possible that cases presenting to the VMTH and the relatively small number of rabbits included in this study may not be representative of the larger population of rabbits presenting to general practitioners for dental maintenance. However, our results emphasize the importance of performing a full oral examination on a regular basis.

The CBCT evaluation of the rabbits in this study demonstrated that oral examination alone is not enough to fully diagnose dental disease. The panoramic image of rabbit #10 (Figures 1A,B) demonstrates how incisor, premolar, and molar occlusion can appear normal, but dental pathology can be present including severe apical elongation, a missing tooth, inflammatory tooth resorption, and moth-eaten pattern of osteolysis of the alveolar bone. The transverse image of rabbit #10 shows a normal 10 occlusal angle on the right and a flat occlusal angle on the left (Figure 1C). This subtle change in occlusal angulation could easily be missed on conscious oral examination. When occlusal angle and dental charting are normal, subtle changes to the dentition can only be detected with advanced imaging. It is established in the literature that some form of imaging should be performed in rabbits with dental disease (1, 4, 8–11); however, this is the first study to recommend the use of CBCT. In feline and canine patients, the diagnostic yield of full-mouth dental radiography has been quantified (21, 22), but this has not yet been performed in rabbits with skull radiography or CT. Our study compared CBCT findings that could be detected on conscious oral examination vs. abnormalities that could not be detected by conscious oral examination. Except for the association between coronal and apical tooth elongation, our results indicate that oral examination findings are poor indicators of additional dental pathology and that diagnostic imaging, such as CBCT, is an essential part of fully characterizing dental disease in rabbits. Our group has separately found CBCT to be superior to conventional CT for evaluation of normal dentition in rabbits (12) and direct comparison of the diagnostic yield of oral endoscopy, skull radiographs, conventional CT, and CBCT should be the basis of future studies investigating rabbit dental disease. Overall, it is important to perform diagnostic imaging in rabbits suspected to have dental disease, despite a normal oral examination.

Malocclusion may be symmetric or asymmetric and the clinical signs may not fully represent the severity of the disease. For instance, rabbit #9 was reported by the client to be eating well and healthy overall but the panoramic image revealed symmetric incisor malocclusion and asymmetric premolar and molar malocclusion (Figures 2A,B). The left maxilla and mandible show apical tooth elongation, coronal tooth elongation, and moth-eaten osteolysis of the alveolar bone. This case illustrates the association between coronal and apical elongation (*P* = 0.029). In contrast, rabbit #2 had relatively mild dental disease but severe clinical signs including decreased fecal output, gastrointestinal stasis, ptyalism, and anorexia. The panoramic image of rabbit #2 depicts an asymmetric premolar and molar malocclusion secondary to a supernumerary right mandibular third premolar tooth (Figures 2C,D). The severity of the apical elongation and the palpable changes to the right ventral mandibular border indicate that this had been a chronic problem, despite the acute nature of the clinical signs. The discrepancy between severity of clinical signs and the extent of underlying dental disease suggests that diagnostic imaging is indicated even for patients with mild clinical signs.

Sharp dental points are an important clinical problem in rabbits and can lead to buccal and lingual ulcers (4, 8, 9). We found the evaluation of dental points *via* CBCT to be difficult, because all maxillary premolar and molar teeth have slightly pointed buccal aspects and mandibular premolar and molar teeth have slightly pointed lingual aspects due to the natural wear of the teeth associated with anisognathism. Although we report 12 of 15 rabbits as having CBCT evidence of sharp dental points, there was poor agreement between CBCT findings and oral examination findings with respect to the presence of sharp dental points. One limitation of our study is that several different clinicians performed oral examinations for the rabbits in this study, and reporting of sharp dental points was not standardized. Variation in the reporting of dental points by different clinicians may have contributed to the poor agreement between CBCT and oral examination for detecting dental points, but it is important to consider that CBCT may be limited in its ability to accurately detect potentially significant dental points.

Although the 3D reconstruction or panoramic images give the clinician a “global feel” of the dentition and maxillofacial features, soft tissue findings can be overlooked if the transverse and custom MPR images are not fully evaluated. For example, the right panoramic image of rabbit #5 shows normal incisor and premolar and molar occlusion (Figure 3A), whereas the sagittal MPR (Figure 3B) and transverse images (Figure 3C) reveal the submandibular swelling, right-sided periocular swelling and chemosis of the nictitating membrane, and gas opacities in the submandibular region, retrobulbar space, and right maxillary first molar germinal center. CBCT scan is optimized to image the osseous maxillofacial features rather than the soft tissue, but evaluation of the soft tissue is still possible. Despite the poorer contrast resolution of CBCT compared to conventional CT, contrast enhancement of small blood vessels in an experimentally induced lesion can be detected following intravenous iodinated contrast administration in rabbits (23). The utility of contrast administration for CBCT of clinical rabbit patients with naturally occurring disease has not yet been described. The improved spatial resolution of CBCT compared to conventional CT may enhance visualization of small, strongly contrast-enhancing lesions and normal structures such as blood vessels, but subtle contrast enhancement may not be detected by CBCT.

One example of subtle findings identifiable on CBCT images is change to the periodontal ligament space. As discussed in part one of this study, CBCT images are significantly superior to conventional CT images for visualizing the periodontal ligament in normal rabbits (*P* < 0.01) (12). In the authors’ experiences, evaluating the periodontal ligament space in multiple planes *via* MPR images is helpful for detection of subtle disease; in particular, the dorsal MPR images allow circumferential visualization of the periodontal ligament width (Figures 4A,C,E). The periodontal ligament should not be evaluated on 3D reconstruction or panoramic images, as previously discussed in part one (12). Periodontitis starts with the inflammation, infection, and widening of the periodontal ligament and loss of the adjacent alveolar bone (5). Figure 4 demonstrates the spectrum of periodontal ligament space changes observed in this study, from subtle periodontal ligament space widening (Figures 4A,B), to periapical lucency suggestive of abscessation (Figures 4C,D), to osteolysis and expansion of the alveolar bone (Figures 4E,F). Given the typical progression of periodontitis to periapical abscessation and osteomyelitis, it is not surprising that periapical lucency was associated with moth-eaten osteolysis of the alveolar bone (*P* = 0.011) in this study. Periapical abscessation and expansion of the alveolar bone can be palpated as a contour change to the ventral mandibular border or present as exophthalmia with decreased ocular retropulsion (24), and detection of these changes on physical examination provide indications that further evaluation of the dentition is necessary.

In areas with loss of alveolar bone secondary to inflammatory conditions such as periodontitis and abscessation, loss of dental substance can occur in the form of inflammatory tooth resorption. Understandably, it was found that periapical lucency (*P* = 0.009), moth-eaten osteolysis of the alveolar bone (*P* = 0.044), and apical elongation (*P* = 0.029) were all associated with inflammatory tooth resorption. Tooth resorption can be difficult to evaluate solely from transverse images, because a tooth that is not fully within the plane of the image can appear as though it is partially resorbed (Figure 5A). Tooth resorption is easier to evaluate on panoramic, sagittal MPR, and dorsal MPR images (Figures 5B–D). Worsening inflammatory resorption can cause tooth fragmentation, making extraction difficult. Without visualization of the fragments, aggressive exploration of the extraction sites could lead to hemorrhage and/or mandibular fracture, as occurred in rabbit #7 mentioned above. Pre-extraction evaluation of the teeth with CBCT can alert the practitioner to the presence of fragments that may complicate extraction.

Previous studies showed preference for the use of transverse CT images for the diagnosis of dental pathology (3, 9, 10, 25); however, in this study, we found that using specialized software to create panoramic, custom MPR, and 3D reconstructed images aided in visualization of specific dental abnormalities. The panoramic and 3D reconstructed images were helpful in evaluating the overall occlusion and the relationship of the teeth to one another. The occlusal angles between the maxillary premolar and molar and the mandibular premolar and molar teeth were visible on transverse images. Transverse images were also useful for delineating the orbit and nasal cavity. The panoramic views provided images of the skull without superimposition of the right and left sides as occurs with skull radiography. Panoramic and sagittal MPR images were useful in evaluating for coronal and apical elongation. To evaluate each individual tooth for dental pathology as well as to assess the soft tissue structures, the transverse and custom MPR images were required. Widening of the periodontal ligament space was more easily identified on dorsal MPR images. Dorsal MPR views also aid in assessment of the orbit, nasal cavity, and sinuses. Inflammatory tooth resorption was more apparent on sagittal and dorsal MPR images and could be confirmed on the transverse images.

The short scanning time, decreased cost, and high-resolution images without the superimposition of anatomic structures make CBCT an appealing method for evaluating rabbit dental disease. The present study is the first to demonstrate the feasibility and yield of CBCT in evaluating maxillofacial features and dentition in rabbits with dental disease. Future studies may compare skull radiography and conventional CT to CBCT in rabbits with dental disease. Independent of the method of imaging used, this study, as well as multiple other studies, substantiate the need to perform advanced imaging when dental disease is suspected. Oral and physical examination is not enough to fully diagnose dental disease.

## Author Contributions

GR: study concept and design; acquisition of data; analysis and interpretation of data; and drafting of manuscript. DC: analysis and interpretation of data; critical revision of the manuscript for important intellectual content; and study supervision. BA: study concept and design; analysis and interpretation of data; critical revision of the manuscript for important intellectual content; administrative, technical, or material support; study supervision; and obtaining funding. DH: critical revision of the manuscript for important intellectual content. PK: statistical analysis. AZ: acquisition of data. FV: study concept and design; analysis and interpretation of data; critical revision of the manuscript for important intellectual content; administrative, technical, or material support; study supervision; and obtaining funding. FV: full access to all of the data in the study and took responsibility for the integrity of the data and the accuracy of the data analysis.

## Conflict of Interest Statement

The authors declare that the research was conducted in the absence of any commercial or financial relationships that could be construed as a potential conflict of interest.
