# Supplementary material for: Clinical Application of Cone Beam Computed Tomography of the Rabbit Head: Part 2—Dental Disease
Source: Front Vet Sci. 2017 Jan 30;4:5. doi: 10.3389/fvets.2017.00005 (PMC5277021; doi:10.3389/fvets.2017.00005)
Supplement: Supplementary file 1 [file Table_1.DOCX]

| **Rabbit #** | **Breed** | **Age (years)** | **Reproductive Status** | **Body weight (kg)** |
| --- | --- | --- | --- | --- |
| 1  2  3  4  5  6  7  8  9  10  11  12  13  14  15 | lop mixed breed  lop mixed breed  Holland lop  California mix  lop mixed breed  Dutch mix  Netherland dwarf  Holland lop  lop  Dutch  lop  mini rex  Dutch  lionhead mix  mini rex | 6.4  6.8  7.8  Adult (estimated 8)  12.2  2.8  5  3.9  2.6  Adult  10.2  1.8  4.6  3.9  8.8 | MC  FS  MC  MC  FS  FI  MC  FS  FS  MI  MC  MI  MC  FS  FS | 1.2  3.2  2.2  2.3  3.2  1.7  1.1  2.4  3.5  1.8  2.4  2.1  1.7  2.2  1.8 |

| **Rabbit #** | **Duration of known dental disease (months)** | **Relevant clinical signs at presentation** | **Reported medical conditions** | **Recent occlusal adjustment** | **Recent dental extractions** | **Recent antibiotic therapy** |
| --- | --- | --- | --- | --- | --- | --- |
| 1 | 0.3 | Hyporexia, ocular discharge, nasal discharge | Dental disease, ascariasis, elevated liver enzymes, emaciation |  |  | X |
| 2 | 11 | Hyporexia, facial swellings/masses, nasal discharge, sneezing | Dental disease, gastrointestinal stasis, mitral valve insufficiency, obesity, pododermatitis, historical uterine and mammary masses | X |  |  |
| 3 | 34.3 | Hyporexia, nasal discharge | Dental disease, otitis externa and bullae osteitis bilaterally, right facial nerve paralysis, left vestibular disease, pododermatitis, facial abscessation |  |  | X |
| 4 | 0.6 | Historical hyporexia, lethargy, and decreased fecal output | Dental disease, recurrent gastrointestinal stasis, pododermatitis |  |  |  |
| 5 | 0.2 | Hyporexia, ocular and nasal discharge, facial swellings/masses, sneezing, exophthalmos | Dental disease, right-sided exophthalmos, suspected laryngitis/tracheitis, pododermatitis, historically elevated liver enzymes |  |  | X |
| 6 | 2.3 | Historical facial swellings/masses | Dental disease, pododermatitis, right retinal scarring |  |  | X |
| 7 | 3 | Ataxia, historical ptyalism | Dental disease, neurologic disease, bilateral otitis media, pododermatitis, chronic sinusitis |  |  |  |
| 8 | 0.3 | Hyporexia, lethargy, nasal discharge | Dental disease, obesity |  |  | X |
| 9 | 2 | No current clinical signs. Patient was presented for maintenance of dental disease. | Dental disease, pododermatitis, obesity, bilateral otitis externa | X |  |  |
| 10 | 0.1 | Facial swellings/masses, ocular discharge, exophthalmos | Dental disease, left-sided exophthalmos, subcutaneous facial abscessation |  |  |  |
| 11 | 62.1 | Ocular and nasal discharge, sneezing | Dental disease, left-sided head tilt, bilateral otitis media and externa |  |  |  |
| 12 | 3.8 | Hyporexia, decreased fecal output, ocular discharge | Dental disease, pododermatitis, moist dermatitis, eyelid mass |  |  | X |
| 13 | 4.2 | No current clinical signs. Patient was presented for maintenance of dental disease. | Dental disease | X |  |  |
| 14 | 4.3 | Hyporexia | Dental disease, pododermatitis | X | X | X |
| 15 | 64.4 | Hyporexia, lethargy | Dental disease, diffuse gingival enlargement, pododermatitis, alopecia |  |  | X |

| **Rabbit Case #** | **Incisor Occlusal Adjustment** | **Premolar-molar Occlusal Adjustment** | **Tooth extractions** | **Marsupialization and/or soft tissue surgery for abscess treatment** | **Bacterial culture and sensitivity** |
| --- | --- | --- | --- | --- | --- |
| **1** | 0 | 0 | 1 | 0 | 1 |
| **2** | 0 | 1 | 0 | 0 | 0 |
| **3** | 0 | 1 | 1 | 0 | 0 |
| **4** | 0 | 1 | 1 | 0 | 1 |
| **5** | 0 | 0 | 0 | 1 | 1 |
| **6** | 0 | 1 | 0 | 0 | 0 |
| **7** | 0 | 0 | 1 | 0 | 1 |
| **8** | 1 | 0 | 1 | 0 | 1 |
| **9** | 1 | 1 | 1 | 0 | 0 |
| **10** | 0 | 0 | 1 | 1 | 1 |
| **11** | 1 | 1 | 0 | 0 | 0 |
| **12** | 1 | 0 | 1 | 0 | 0 |
| **13** | 0 | 1 | 0 | 0 | 0 |
| **14** | 1 | 1 | 1 | 0 | 0 |
| **15** | 1 | 1 | 0 | 0 | 0 |
| **Total** | 6 | 9 | 9 | 1 | 6 |
